# Central Giant Cell Granuloma of the Mandible and Maxilla: A Clinicopathological Study of 21 Cases

**DOI:** 10.7759/cureus.63043

**Published:** 2024-06-24

**Authors:** Ahmed Lazim, FNU Sakshi, Samir M Amer, Vinay S Mallikarjuna, Dina Zenezan, Riya Kuklani, Daniela Proca

**Affiliations:** 1 Pathology, Temple University, Philadelphia, USA; 2 Pathology, PathGroup, Nashville, USA; 3 Pathology and Laboratory Medicine, Temple University Hospital, Philadelphia, USA; 4 Pathology, University of Cincinnati Medical Center, Cincinnati, USA; 5 Pathology, Temple University Hospital, Philadelphia, USA

**Keywords:** intraosseous, giant cells, maxilla, mandible, central giant cell granuloma

## Abstract

Background

Central giant cell granuloma (CGCG) presents as a locally invasive, intraosseous lesion characterized by the accumulation of multinucleated giant cells amidst a matrix of hemorrhage and reactive fibrous tissue that infiltrates bone trabeculae. This idiopathic non-neoplastic proliferative lesion primarily affects the mandible, typically presenting as either unilocular or multilocular radiolucencies on X-rays. Although trauma or intraosseous hemorrhages are potential triggers, the precise histogenesis and etiology remain unclear. CGCG predominantly occurs in children and young adults, with a slight female predilection.

Methods and materials

A retrospective analysis of 21 cases of CGCG diagnosed at the Oral Pathology/Pathology department of Temple University Hospital between 2015 and 2022 was conducted. Each case was evaluated based on various parameters, including age, gender, presenting symptoms, radiographic findings, clinical differential diagnosis, and histological confirmation. The primary radiographic technique employed for diagnosis was X-ray imaging of the mandible and maxilla. The histological examination involved cutting paraffin-embedded tissue into 5-micrometer-thick sections, which were then stained using routine hematoxylin and eosin (H&E) stain. Notably, no specialized histochemical or immunohistochemical stains were utilized in the evaluation process.

Results

In our study, we reviewed 21 cases; 9 were male, 11 were female, and one had no available gender data. The age range was 15-76 years, with a mean of 50 years. The mandible was the most commonly affected location (17 cases; 81%) while the maxilla was less commonly involved (4 cases; 19%). Many CGCG lesions were asymptomatic (13 cases; 62%); eight cases (38%) were symptomatic, with pain and fullness of the affected dental region being the main manifestations. In a few cases, conditions such as brown tumor (severe hyperparathyroidism) and odontogenic neoplasms, such as ameloblastoma, were suspected clinically and radiographically. The diagnosis of CGCG with associated acute and chronic inflammation was confirmed in all the cases. Histological evaluation of routinely stained slides was the main diagnostic tool utilized. No special stains or molecular studies were required to establish the final diagnosis.

Conclusions

Our investigation has determined that CGCG exhibits a non-neoplastic nature, displaying a spectrum of behaviors ranging from non-aggressive to aggressive tendencies. While CGCG is predominantly observed in the mandible, rare instances of involvement in the maxilla have also been documented. Importantly, no confirmed association with neoplastic lesions was identified during our analysis. The clinical course of CGCG tends to be indolent, with some cases presenting in association with impacted teeth. It's noteworthy that CGCG can present features mimicking neoplastic conditions, such as ameloblastoma, or localized lesions linked to systemic disorders such as hyperparathyroidism (brown tumor).

## Introduction

Central giant cell granuloma (CGCG) is a benign but locally aggressive lesion that occurs in the mandible and maxilla. CGCG was first described by Jaffe in 1953 [1]. Histologically, CGCG is characterized by the proliferation of monocytic/spindle mesenchymal cells with aggregates of multinucleated osteoclast-type giant cells, in a background of hemorrhage and reactive fibrous tissue infiltrating the eroded bone trabeculae [1,2]. The exact etiology of CGCG remains unknown, but it is thought to be a reactive lesion to local trauma, infection, or hormonal factors [1-4]. Radiographically, CGCGs appear as unilocular or multilocular radiolucent lesions, more commonly in the mandible (70%). Children and young adults are more commonly affected, with a slight female prevalence [4-6]. There is a 10% risk of recurrence and some GCCG may exhibit aggressive behavior, necessitating enucleation, and curettage or osteotomy for complete removal of the lesion [2,3,6,7]. Non-surgical treatment with alpha interferon (alpha-IFN), calcitonin, and corticosteroids has been described in the literature [8-11]. The purpose of this case series is to review the clinical, radiographic, and histopathological features of CGCG of the mandible and maxilla.

This paper was presented as a poster presentation at the USCAP 112th Annual Meeting. Laboratory Investigation (2023 Suppl), 103(3): 1001-1062. It is also uploaded as a Preprint on Researchsquare: https://www.researchsquare.com/article/rs-3459321/v3 on 28 February 2024.

## Materials and methods

A comprehensive retrospective search was conducted within the Temple University Hospital's pathology electronic database, covering the period from January 1, 2015, to December 31, 2022. The study included patients aged 18 to 99 years who received a pathological diagnosis of CGCG through biopsy or excision. In total, 21 cases of CGCG diagnosed by the oral pathology service at our institution over the preceding seven-year period were identified and analyzed for clinicopathological parameters, including age, gender, symptoms, anatomical site, radiographic findings, clinical presentation, differential diagnosis, and histopathologic diagnosis. Cases lacking complete clinical, radiographic, or histopathologic data were excluded. This series included biopsy specimens sent from referral hospitals to our Oral Pathology service for pathological assessment. The diagnostic process involved examining available clinical and radiologic histories (as documented in the pathology reports) and assessing hematoxylin and eosin (H&E) stained sections, with each slide reviewed at magnifications of 100x, 200x, and 400x. This thorough review culminated in the confirmation of the definitive diagnosis. Unfortunately, long-term follow-up data were not available for many of the reviewed CGCG cases.

## Results

Twenty-one cases were evaluated with an established histopathologic diagnosis of CGCG. The demographics included 9 males and 11 females; a single case was of unknown gender, revealing slightly increased prevalence in the female population. The age of the patients in our cohort ranged from 15-76 years, with a mean of 49.5 years (standard deviation 21.35). For the anatomic site, the majority of our cases demonstrated mandibular involvement (n=17; 81%) while the remaining (n=4, 19%) showed maxillary involvement. Most of our cases were asymptomatic (n=13; 62%), with few cases (n=8; 38%) showing symptoms due to expansile lesions and cortical bone resorption. Radiographic findings, whenever available, were reviewed. Radiographically, most cases showed well-defined, radiolucent lesions (n=16) or mixed radiolucent and radiopaque patterns (n=1). In our study, four cases diagnosed with CGCG showed impacted tooth/teeth, whereas expansile lesions and cortical bone resorption were other radiologic features noted (Table 1).

**Table 1:** Clinicopathologic characteristics and radiographic findings of 21 cases of central giant cell granuloma M: male; F: female; NA: no information available; LT: left; RT: right; no: number; CGCG: central giant cell granuloma; DDX: differential diagnosis. *Differential diagnoses are provided in the referral requisition forms.

Case	Age (years)	Sex	Location	Clinical presentation	Radiographic findings	DDX*	Microscopic diagnosis
1	54	M	LT anterior mandible	Asymptomatic	NA	NA	CGCG
2	19	F	RT posterior mandible	Asymptomatic	Radiolucent	NA	CGCG
3	39	F	LT anterior mandible	Numbness	Radiolucent	Hyperparathyroidism	CGCG
4	45	F	Mandible	Submental fullness with mucocutaneous fistula	NA	NA	CGCG
5	61	M	LT posterior mandible	Asymptomatic	Radiolucent	NA	CGCG and abscess
6	74	F	LT posterior mandible	Asymptomatic	Radiolucent	NA	CGCG
7	67	M	Anterior mandible	Asymptomatic	Radiolucent	NA	CGCG
8	76	M	LT posterior maxilla	Pain and fullness	Mixed radiolucent and radiopaque	NA	CGCG and chronic sinusitis
9	15	F	RT posterior mandible	Asymptomatic	Radiolucent with impacted tooth no. 32	NA	CGCG
10	16	M	RT posterior mandible	Asymptomatic	Radiolucent, expansile	NA	CGCG
11	59	M	LT anterior mandible	Asymptomatic	Radiolucent lesion causing cortical bone resorption	NA	CGCG
12	68	F	LT anterior mandible	Pain and fullness	Radiolucent	NA	CGCG
13	17	F	RT posterior mandible	Pain and fullness	Radiolucent with impacted teeth no. 31 & 32	NA	CGCG
14	60	M	RT maxilla	Asymptomatic	Radiolucent	NA	CGCG
15	57	F	LT anterior maxilla	Buccal bony expansion of area of no. 11	NA	NA	CGCG
16	54	M	RT posterior maxilla	Pain	NA	NA	CGCG
17	39	F	RT posterior mandible	Asymptomatic	Radiolucent	Ameloblastoma, lateral periodontal cyst	CGCG
18	71	M	RT posterior mandible	Pain and fullness	Radiolucent with impacted tooth no. 31	Granuloma, abscess, cyst	CGCG
19	71	F	LT mandible	Asymptomatic	Radiolucent	Ameloblastoma	CGCG
20	16	F	RT mandible	Asymptomatic	Radiolucent with impacted teeth no. 26 &27	NA	CGCG
21	62	NA	Mandible	Asymptomatic	Radiolucent (multilocular)	NA	CGCG

The clinical and radiologic differentials, as provided in the referral requisition forms accompanying each specimen, encompassed a spectrum of conditions ranging from benign entities like chronic granulomatous inflammation, acute inflammatory processes/abscess formation, and brown tumor (associated with hyperparathyroidism), to rarer occurrences such as non-ossifying fibroma, as well as neoplastic entities including odontogenic neoplasms such as ameloblastoma. Other neoplastic entities such as giant cell tumor of bone (GCT), osteoblastoma, aneurysmal bone cyst (ABC), and chondrosarcoma, while not specifically mentioned in the referral forms, should also be considered among the potential differential diagnoses. This spectrum of differential diagnoses aligns with those discussed in the relevant literature [12-14].

**Figure 1:**
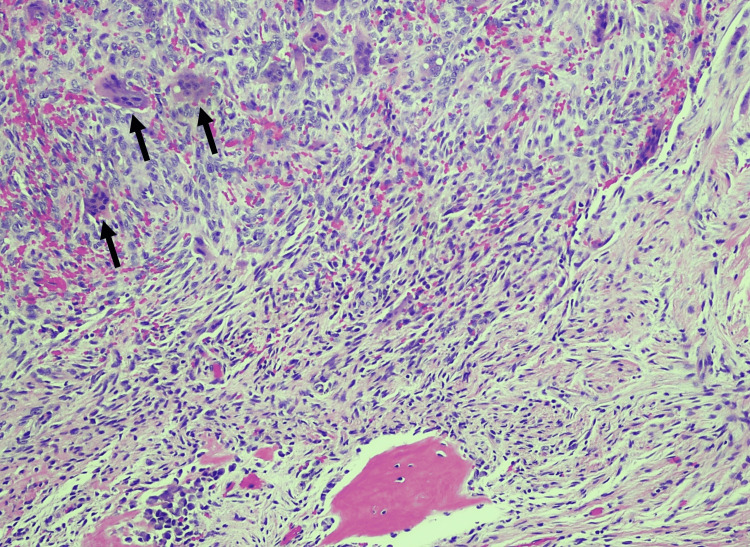
Classical morphology of central giant cell granuloma (CGCG) Numerous multinucleated giant cells (marked by black arrows), fibroblasts, and reactive bone formation, accompanied by extravasated red blood cells (Hematoxylin & Eosin stain, 100x).

**Figure 2:**
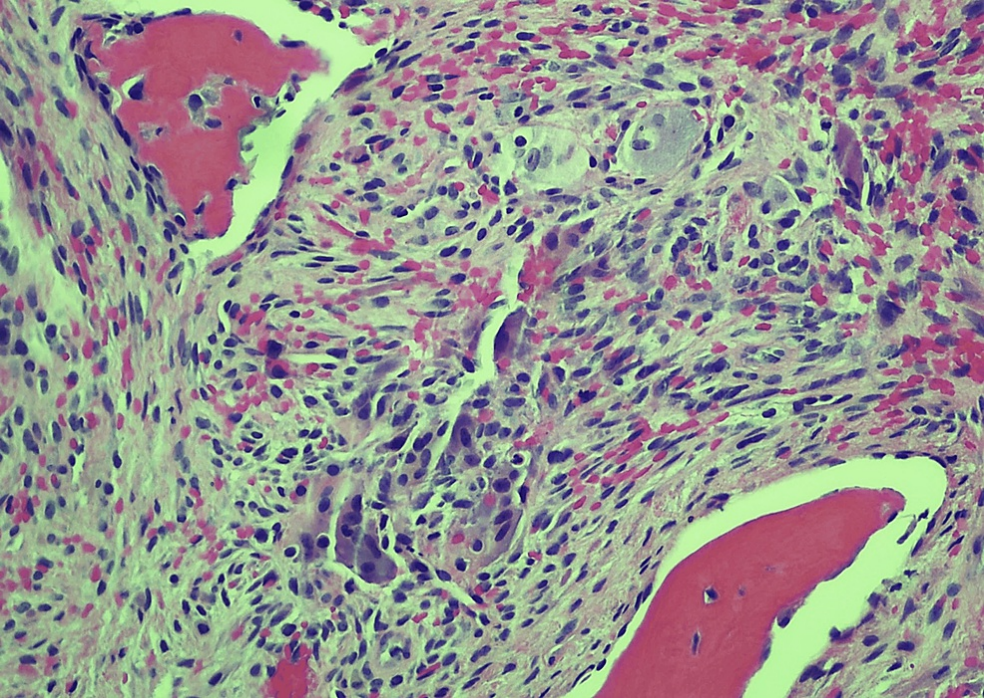
Central giant cell granuloma (CGCG) with higher magnification, focused on bland spindled cells, multinucleated giant cells, reactive trabecula bone, and scattered red blood cells Hematoxylin & Eosin stain, 200x

Upon histopathological examination of H&E-stained slides, characteristic features were observed, including the presence of numerous multinucleated giant cells, fibroblasts, and reactive bone formation, accompanied by extensive red blood cell extravasation and droplets of hemosiderin pigmentation (Figures 1-2). The multinucleated giant cells typically exhibited central localization within the lesion or were dispersed throughout, often featuring nuclei ranging from a few to more than 20, in concordance with previously described cases [12,13]. The fibroblasts, appearing as spindle-shaped cells, demonstrated a disorganized arrangement, contributing to collagen fiber production. Reactive bone formation was observed either at the lesion's periphery or within its confines. Importantly, the histopathological analysis served as a definitive tool in confirming the diagnosis across all cases, particularly in scenarios where the clinical differential encompassed a wide array of benign and neoplastic conditions.

## Discussion

CGCG typically presents as a painless swelling or mass that gradually increases in size in non-aggressive lesions. However, in aggressive cases, the lesion may be associated with pain, root resorption, paresthesia, and tooth mobility, contingent upon its location and size. The age of onset of CGCG is usually between 10 and 30 years, with a higher incidence among females. Radiographically, the majority of cases in our study presented as a well-defined, radiolucent lesion with variable borders. It may exhibit unilocular or multilocular patterns and can cause root resorption or displacement of teeth [2-7]. The radiographic finding of the multilocular pattern should prompt consideration of other potential differential diagnoses, including odontogenic keratocyst (OKC), ameloblastoma, hemangioma/arteriovenous malformation (AVM), odontogenic myxoma, and botryoid odontogenic cyst [4]. Occasionally, the lesion may appear as a mixed radiolucent-radiopaque entity, attributed to the presence of calcifications or ossifications within the lesion [4-7].

The histopathological features of CGCG typically include the presence of osteoclast-like multinucleated giant cells, spindle-shaped fibroblast-like stromal cells, and round mononuclear cells. The giant cells are usually located in the central part of the lesion and contain numerous nuclei with prominent nucleoli. Spindle-shaped fibroblasts are distributed throughout the lesion and are responsible for collagen fiber production [2]. Reactive bone formation may be seen at the lesion's periphery or within it. Additionally, CGCG often features hemosiderin-laden macrophages, and extravasated red blood, which can sometimes prompt consideration of differential diagnoses with GCT, ABC, and brown tumors of hyperparathyroidism. The correlation of both radiographic and histologic features can aid in discerning the clinicopathologic behavior of the lesion as either aggressive or non-aggressive [2].

The pathogenesis of CGCG is not fully understood though it is believed to stem from a reactive process triggered by local factors such as trauma, infection, or hormonal influences. Some studies suggest that CGCG is a result of an abnormal immune response, where the multinucleated giant cells represent activated macrophages and osteoclast-like cells [12-16]. Studies have shown that CGCG expresses markers of osteoclastogenesis such as CD68, RANK (Receptor Activator of Nuclear Factor Kappa-B (NF-κB)), RANKL (Receptor Activator of Nuclear Factor Kappa-B (NF-κB) Ligand), and OPG (Osteoprotegerin). These markers play roles in regulating osteoclast differentiation and activation, implying an association between CGCG and osteoclastic activity [17].

Giant cell lesions of the jaws may rarely occur in the setting of RASopathy syndromes such as Noonan syndrome or neurofibromatosis. Recently, CGCG has been recognized as benign neoplasms characterized by Ras/MAPK signaling pathway mutations [16,17].

Chrcanovic et al. demonstrated that sporadic CGCG of the jaws do not share the H3F3A pGly34Trp or p.Gly34Leu mutations reported in GCT occurring in long bones. These findings contribute to the growing body of evidence suggesting that CGCG of the gnathic bone is distinct and separate from the extragnathic GCT [18,19].

In summary, CGCG is a rare intraosseous, non-neoplastic, locally aggressive lesion that typically presents as a painless swelling or mass in the mandible and maxilla. It features multinucleated giant cells, fibroblasts, and reactive bone formation. CGCGs are distinct from long bone GCT. Despite being considered reactive, its histogenesis and etiology remain unknown. The combined radiographic and histopathological features of CGCG are somewhat specific for the experienced diagnostician. Recent studies have identified genetic alterations in CGCG, which may contribute to the dysregulation of osteoclastogenesis and explain clinical behavior, while at the same time may be useful in diagnosing this entity. Treatment strategies vary based on lesion size, location, and aggressiveness, ranging from observation for small lesions to surgical intervention, including curettage, enucleation, or resection for larger ones. Recurrence rates are higher for aggressive or multilocular lesions, particularly in the mandible. Long-term follow-up is crucial for monitoring recurrence. Further research is imperative to deepen our understanding of CGCG's etiology and pathogenesis and to develop more efficacious treatments.

Limitations of the study

One limitation of this study is the relatively small number of CGCG cases (21), which may not be representative of the broader population and could affect the robustness and generalizability of the findings. Additionally, markers of osteoclastogenesis such as CD68, RANK, RANKL, and OPG have not been analyzed in this case series.

## Conclusions

The accurate diagnosis of CGCG heavily relies on thorough histopathologic evaluation, as clinical and radiological assessments often present a broad spectrum of potential differential diagnoses. Given the complexity of interpretation and the limitations inherent in small biopsy samples, it is imperative to underscore the significance of raising awareness regarding this entity. In cases where uncertainty arises, seeking a second opinion from an experienced oral pathologist becomes crucial to ensure an accurate diagnosis and mitigate the risk of misclassification as a neoplasm. This collaborative approach not only improves diagnostic accuracy but also promotes better patient outcomes by facilitating the implementation of appropriate management strategies.
